# Evaluation of Functional Electrospun Chitosan-Based Nanofibers Loaded with Norfloxacin for Enhanced Burn Wound Healing Response

**DOI:** 10.3390/polym18131621

**Published:** 2026-06-30

**Authors:** Corneliu-George Coman, Ioannis Gardikiotis, Carmen Solcan, Cosmin-Gabriel Tartau, Caroline Chabot, Gianina Dodi, Liliana Mititelu Tartau

**Affiliations:** 1 Grigore T. Popa University of Medicine and Pharmacy, 700115 Iasi, Romania; dr.cgcoman@gmail.com (C.-G.C.); cosmin.tartau@gmail.com (C.-G.T.); gianina.dodi@umfiasi.ro (G.D.); liliana.tartau@umfiasi.ro (L.M.T.); 2 Department of Surgery, Faculty of Medicine, Pharmacy and Biomedical Sciences, University of Mons, 7000 Mons, Belgium; 3 Sfanta Maria Children’s Emergency Hospital Iasi, 700309 Iasi, Romania; 4 Faculty of Veterinary Medicine, Ion Ionescu de la Brad University of Life Sciences, 700490 Iasi, Romania; carmensolcan@yahoo.com; 5 Department of Radiology, Cliniques Universitaires Saint Luc, Université Catholique de Louvain, 1200 Brussels, Belgium; caroline.chabot@saintluc.uclouvain.be

**Keywords:** electrospun nanofibers, trimethyl chitosan, norfloxacin, wound healing, rat burn model

## Abstract

Nanofibrous materials based on chitosan (CS) have attracted considerable attention for advanced wound management due to their excellent biocompatibility and their suitability as drug delivery systems for wound healing applications. Additional surface modification may improve their interaction with the wound environment and influence tissue repair mechanisms. TMC/CS nanofibers were fabricated via electrospinning and subsequently processed into three formulations: unloaded fibers (NCC), norfloxacin-loaded fibers (NCX), and norfloxacin-loaded fibers modified with 2-formylphenylboronic acid (NCXA). The resulting materials were characterized using scanning electron microscopy (SEM), Fourier-transform infrared spectroscopy (FTIR), thermogravimetric analysis (TGA), and UV–Vis spectroscopy. Their therapeutic performance was evaluated in a standardized deep dermal burn model in *Wistar* rats, with Vaseline gauze and silver sulfadiazine serving as reference treatments. Wound healing progression was assessed through macroscopic examination, histopathological analysis, immunohistochemical evaluation of TNF-α, IL-1β, IL-17, VEGF, VCAM, and CD163 expression, and systemic IL-8 determination. Physicochemical characterization confirmed homogeneous nanofiber formation, efficient incorporation of norfloxacin, and successful surface modification. All electrospun formulations promoted improved healing outcomes compared with the untreated control group. Among them, the norfloxacin-loaded nanofiber formulation demonstrated the most pronounced wound-healing effect, characterized by faster re-epithelialization, attenuation of inflammatory mediators during later healing stages, and superior tissue architecture restoration. Conversely, the 2-formylphenylboronic acid-modified norfloxacin-loaded fiber formulation maintained a more persistent inflammatory state and exhibited a slower transition into the remodeling phase. Trimethyl chitosan-based nanofibers loaded with norfloxacin show strong potential as multifunctional wound dressing platforms capable of controlled drug release. The findings indicate that formulation composition plays a critical role in regulating inflammation and tissue regeneration, underscoring the need for continued refinement of chitosan-derived nanosystems for burn wound therapy.

## 1. Introduction

Burn injuries represent one of the most significant challenges in modern healthcare, affecting millions of individuals worldwide and generating substantial medical and socioeconomic burden. Their management is often complicated by the prolonged healing period, the high risk of infection, and the extensive resources required for continuous wound monitoring and care. In addition to the direct clinical impact, burn treatment involves considerable financial costs related to hospitalization, repeated interventions, specialized therapies, and the daily replacement of wound dressings [1]. The multifactorial nature of burn wound healing, which includes inflammation control, prevention of microbial contamination, pain reduction, and stimulation of tissue regeneration, underlines the necessity for more efficient and patient-friendly therapeutic approaches [2].

Frequent dressing replacement remains one of the most distressing aspects of burn care. These repeated procedures may intensify the initial trauma by causing additional tissue damage and severe discomfort, often requiring repeated administration of general anesthesia, particularly in patients with extensive injuries [1,2]. Beyond the immediate physical suffering, recurrent painful interventions can also contribute to long-term psychological complications, including anxiety, post-traumatic stress disorder, and persistent chronic pain syndromes. Consequently, improving dressing performance and reducing the frequency of dressing changes have become important objectives in the development of modern burn therapies.

In recent years, bioengineered wound dressings have emerged as promising alternatives to conventional materials due to their ability to create a favourable microenvironment for healing while simultaneously delivering antimicrobial and regenerative agents [3]. Such advanced systems are increasingly being investigated as potential next-generation solutions for burn wound management, offering enhanced biocompatibility, improved moisture balance, controlled therapeutic delivery, and reduced patient discomfort [1,2,3].

**Figure 1:**
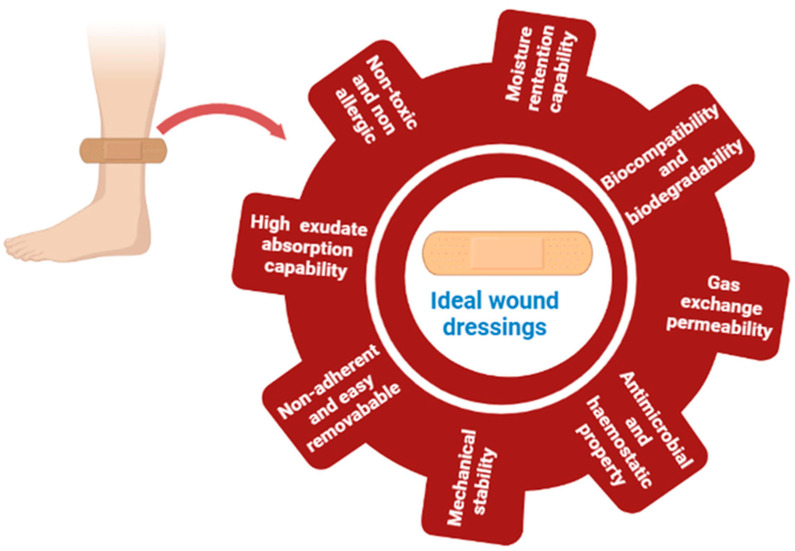
Ideal dressing characteristics.

An effective wound dressing (schematically presented in Figure 1) should combine multiple functional characteristics that actively support the healing process. Besides providing mechanical protection and creating a controlled barrier against external contaminants [4], modern dressings are expected to maintain an optimal wound microenvironment and adapt dynamically to the changing physiological conditions that occur throughout the different stages of tissue repair [5,6,7,8]. Such multifunctional systems are particularly important in burn care, where the healing process is frequently complicated by excessive inflammation, microbial colonization, and impaired tissue regeneration.

Among the biomaterials explored for advanced wound management, chitosan (CS) has gained considerable interest due to its favourable biological and physicochemical properties.

Derived from chitin, chitosan is a biodegradable and biocompatible polysaccharide known for its intrinsic antibacterial, antiviral, and antifungal activities, as well as its hemostatic capacity and ability to absorb wound exudates. These features make CS especially attractive for the development of burn wound dressings and regenerative biomaterials [3,9,10,11]. Furthermore, chemically modified water-soluble derivatives of chitosan, such as N,N,N-trimethyl chitosan chloride (TMC), exhibit improved solubility over a broad pH range together with enhanced antimicrobial performance, thereby expanding their biomedical applicability [12,13,14].

An additional advantage of chitosan-based matrices lies in their ability to incorporate and release therapeutic agents through physical interactions or chemical bonding mechanisms [15,16]. Norfloxacin is a second-generation fluoroquinolone antibiotic (molecular weight: 319.33 g/mol) with broad-spectrum activity against both Gram-positive and Gram-negative microorganisms. The molecule exhibits amphoteric properties due to the presence of carboxylic and piperazine groups, which influence its solubility and interactions with polymeric matrices. Although norfloxacin displays limited aqueous solubility at physiological pH, its physicochemical characteristics make it particularly suitable for incorporation into controlled-release systems, where localized delivery can enhance drug retention and maintain therapeutic concentrations at the wound site [15,17,18]. Moreover, its multiple functional groups promote intermolecular interactions with chitosan-based carriers, facilitating drug loading and sustained release [15,16]. Clinically, norfloxacin has been widely used for the treatment of urinary tract and other susceptible bacterial infections. However, the systemic use of fluoroquinolones has become increasingly restricted due to concerns regarding adverse effects, including tendinopathy, neurological complications, and the potential development of bacterial resistance. Consequently, localized delivery approaches have attracted growing interest as a strategy to preserve the antimicrobial efficacy of norfloxacin while minimizing systemic exposure and associated side effects. In this context, norfloxacin represents a promising candidate for localized antimicrobial therapy and wound infection management. Previous studies have demonstrated a synergistic antibacterial effect between norfloxacin and chitosan, highlighting the potential of this combination for advanced wound dressing applications [19,20,21,22].

Despite the promising antimicrobial activity of norfloxacin, currently available topical formulations still present several important limitations. Conventional creams, gels, and ointments often exhibit poor residence time at the wound surface, require frequent reapplication, and provide limited control over drug-release kinetics. In addition, rapid drug diffusion and insufficient interaction with the wound bed may result in suboptimal local concentrations, particularly in highly exudative burn wounds. These shortcomings have stimulated the development of advanced drug-delivery platforms capable of protecting the active compound, prolonging its retention at the injury site, and ensuring sustained antimicrobial activity throughout the healing process [17,23,24].

Recent progress in nanotechnology and scaffold engineering has further improved the performance of chitosan-derived dressings. In particular, nanostructured systems possessing pH-responsive behavior and a high liquid absorption capacity can interact dynamically with the wound environment, making them suitable for the treatment of ulcerative lesions and moderate-to-severe burns [10,25,26]. Building upon these advances, several studies have focused on the development and characterization of a novel patented chitosan-based dressing with unique structural and functional features [24,27,28,29,30]. Boronic acid derivatives have attracted increasing interest in biomedical applications due to their ability to participate in dynamic covalent interactions and modulate material surface properties. Although chitosan-based nanofibrous dressings have demonstrated considerable promise for wound management, limited information is available regarding the biological consequences of combining norfloxacin loading with additional surface modification strategies in burn wound healing. In particular, the impact of such modifications on inflammation resolution, tissue regeneration, angiogenesis, and overall healing quality remains insufficiently understood. Therefore, the present study aimed to develop electrospun TMC/CS nanofibers loaded with norfloxacin and to investigate the effects of subsequent modification with 2-formylphenylboronic acid. The therapeutic performance of the resulting formulations was evaluated in a standardized rat burn model through comprehensive physicochemical characterization, macroscopic assessment, histological examination, immunohistochemical analysis, and systemic inflammatory profiling.

## 2. Materials and Methods

### 2.1. Materials

All reagents were obtained from Sigma-Aldrich Chemical Co. (Steinheim, Germany) and utilized without further purification. Chitosan (127 kDa, 97% degree of deacetylation) was synthesized via alkaline hydrolysis of low-molecular-weight chitosan. Other chemicals employed included acetic acid (99.89%), norfloxacin (98%), poly(ethylene oxide) (PEO, 1000 kDa), 2-formylphenylboronic acid (97%), sodium hydroxide (95%), and ethanol (98.89%).

### 2.2. Preparation and Characterization of Nanofibers

N,N,N-Trimethyl chitosan (TMC) was synthesized according to a previously reported protocol without modification [31]. Briefly, a TMC/CS/PEO composite solution (7:1:2 mass ratio) prepared in 75% acetic acid was electrospun under controlled conditions using an NanoSpinner StarterKit (Inovenso, Istanbul, Turkey) (12 kV applied voltage, 0.4 mL h^−1^ flow rate, 10 cm needle-to-collector distance, 0.8 mm needle inner diameter, 800 rpm collector rotation speed, and a temperature of 27–28 °C). The resulting nanofibrous mats were washed with dehydrated absolute ethanol, previously stored over molecular sieves, to remove the sacrificial polymer (PEO). The washed mats were subsequently dried at room temperature under ambient conditions until a constant weight was reached.

The nanofibers were then processed into three formulations: unloaded fibers (NCC), norfloxacin-loaded fibers (NCX), and norfloxacin-loaded fibers further modified with 2-formylphenylboronic acid (NCXA). Norfloxacin loading was achieved by equilibrium adsorption through immersion of the nanofibers in a 0.1% ethanolic norfloxacin solution for 24 h. Surface functionalization was subsequently performed by spraying the norfloxacin-loaded fibers with a 0.2% ethanolic solution of 2-formylphenylboronic acid. The amount of aldehyde was adjusted to obtain a glucosamine-to-aldehyde molar ratio of 10:1 [32]. No additional washing steps were applied after norfloxacin loading or surface modification to prevent the loss of adsorbed drug molecules and aldehyde-containing surface functionalities. After each processing step, the samples were dried at room temperature under ambient conditions until constant weight.

Dual TMC/CS nanofibers were fabricated using PEO as a sacrificial agent, subsequently removed by ethanol treatment [33]. Norfloxacin, a broad-spectrum fluoroquinolone antibiotic, was incorporated by immersion in ethanolic solution, resulting in a loading degree of 3.53 ± 0.95%. The drug-loaded fibers were further functionalized through the formation of reversible imine bonds with 2-formylphenylboronic acid, a compound known for its antimicrobial, antifungal, and antioxidant properties [34,35].

The nanofibers were characterized using complementary physicochemical techniques. Fourier-transform infrared (FTIR) spectra were recorded in ATR mode using a VERTEX 70 FT-IR spectrophotometer (Bruker, Karlsruhe, Germany) over the range of 4000–600 cm^−1^, with 32 scans acquired at a resolution of 4 cm^−1^. Spectral processing was performed using OPUS 6.5 software. Fiber morphology was examined by field-emission scanning electron microscopy (FE-SEM; EDAX Quanta 200, Thermo Fisher Scientific, Waltham, MA, USA) operated at an accelerating voltage of 20 kV. Prior to imaging, samples were sputter-coated with gold to improve surface conductivity. Fiber diameters were determined from SEM micrographs using ImageJ 1.54 software. Morphological analysis was additionally performed by polarized optical microscopy (Axio Imager M2, Zeiss, Jena, Germany) under crossed polarizers.

Norfloxacin loading was quantified by UV–Vis spectroscopy (Cary 60, Agilent Technologies, Santa Clara, CA, USA) at 272 nm using the Beer–Lambert law and a calibration curve described by the equation *y* = 0.0478*x* − 0.0426 (R^2^ = 0.9959), where *y* corresponds to absorbance and *x* to norfloxacin concentration. The loading degree (LD, %) was calculated as *LD* = (*m*/*m*_0_) × 100, where *m* is the mass of norfloxacin incorporated into the nanofibers and *m*_0_ is the initial mass of the sample (5 mg).

Thermal stability was evaluated by thermogravimetric analysis (TGA) using an STA 449 F1 Jupiter analyser (Netzsch, Selb, Germany) equipped with a 100 μL platinum crucible. Measurements were performed from 30 to 600 °C at a heating rate of 10 °C min^−1^.

### 2.3. Animals

Experimental procedures were performed on healthy, non-genetically modified, specific pathogen-free adult male *Wistar* rats (300–350 g) obtained from the Cantacuzino National Institute for Research and Development (Băneasa Station, Bucharest, Romania). Animals were acclimated for 7 days under controlled environmental conditions: temperature 21 °C ± 2 °C, relative humidity 50 ± 5%, and a 12 h/12 h light/dark cycle. Standardized food pellets and water were provided ad libitum.

The study was carried out at the Grigore T. Popa University of Medicine and Pharmacy Iași at Ostin C. Mungiu Advanced Research and Development Center for Experimental Medicine, with standardized environmental conditions and consistent animal welfare practices throughout the experimental period. The animal study protocol was approved by the Ethical Committee of the university (certificate no. 160/04.03.2022 and Project authorization no. 56/19.02.2022). The study was conducted in strict accordance with applicable national and international ethical guidelines for the care and use of laboratory animals, including the 3R principles of the European Directive 2010/63/EU and Romanian Law No. 43/2014, ensuring the welfare and humane treatment of all animals throughout the study.

### 2.4. In Vivo Burn Protocol

**Figure 2:**
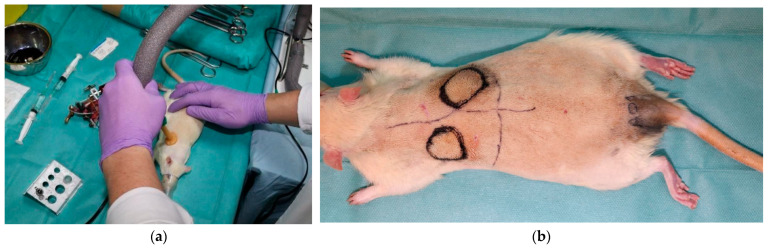
(**a**) Experimental setup for rat preparation. Burn infliction funnel is placed on shaved rat dorsum; (**b**) Burns with tattooed contour and tattooed rat code at the base of the tail. Skin marker used for bony landmarks.

On Day 0, all animals were anesthetized through isoflurane, weighed, and the hair was shaved on their lateral and dorsal regions. Burns were inflicted using the author’s patented original device [36] (Figure 2) by ejecting distilled water vapors at 100 °C (212 °F) on the clean, hair-free rat’s back skin.

The model generated two burn sites on either side of the dorsal thoracic region, separated by a minimum of 1 cm of intact skin. The protocol, previously validated in earlier studies [37,38,39,40], involved a 2-s vapor exposure, which showed to produce consistently deep partial-thickness (Figure 2b) burns [10,39,40,41]. The burn margins were precisely delineated by tattooing the surrounding intact skin with black inorganic ink using a standard tattoo device.

The animals were divided into 5 groups (5 animals/group) for the 3 types of fibers, namely NCC, NCX and NCXA, 1 for the positive control receiving a thin (≈1 mm) layer of silver sulfadiazine cream (SS) and 1 for the negative control treated with petroleum jelly (V). Each animal had 2 burns on their back, covered with each material, both covered with four circular gauze pads followed by a patented wound dressing device, as explained in the next section. Post-burn analgesia was ensured by intraperitoneal administration of tramadol (20 mg/kg) and continued in the drinking water for a minimum of 3 days [42,43,44,45], according to visual rat grimace pain scale.

### 2.5. Patented Wound Dressing

The patented wound dressing is engineered with a circular base fabricated from 3D-printed PLA, incorporating six banana-shaped piercings designed to facilitate controlled dressing change and a commercially available bi-stable metal lid.

**Figure 3:**
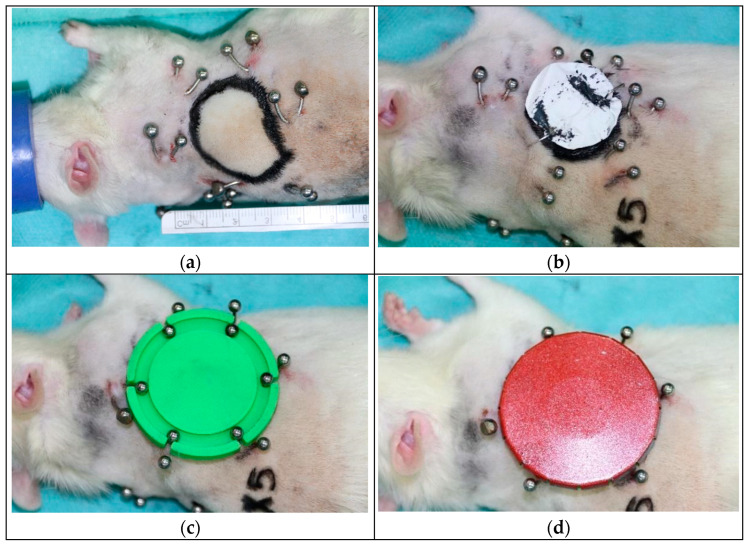
Dressing assembly procedure. (**a**) Piercing placement (60 degrees, equally spaced); (**b**) Chitosan dressing applied; (**c**) Gauze and plastic protection. Piercing balls in their respective slits; (**d**) Ensemble fixation under the metallic bistable lid.

The dressing is completed with a lid featuring a depressible top, enabling precise application and solid retention of therapeutic agents within the dressing chamber [46]. The overall architecture and functional components are depicted in Figure 3, demonstrating the innovative integration of mechanical and biomedical design features.

### 2.6. Follow-Up and Evaluation Protocols

Rats were monitored daily throughout the study, with no significant distress observed. Dressings were applied on day 0 and day 11 only. Digital photographs of the wounds were taken at three time points: immediately after burn induction (day 0), day 11, and day 22. Biopsies were collected on days 11 and 22 under general anaesthesia. Wound areas were quantified by digital planimetry using ImageJ software, and statistical analyses were conducted with SPSS (version 17.0). The study endpoint was defined as complete epithelialization of all wounds within a given group. Euthanasia was performed under general anesthesia via intracardiac injection of 2 mL KCl after the collection of target organs (liver, kidney, lymph nodes, tendons) and blood samples. All procedures were conducted in accordance with the national and international standards for animal care [42,45,47,48].

The collected tissue samples were embedded in paraffin, sectioned using a microtome, and stained with haematoxylin-eosin (H&E, Baton Rouge, LA, USA) and immunohistochemically for VEGF, TNF-α, IL-1β, IL-17, VCAM, and CD163 (Bio-Rad Laboratories, Hercules, CA, USA). Histological examination was performed with an optical microscope equipped with a digital imaging system (Nikon E600 Eclipse, Tokyo, Japan) [49,50,51,52]. Wound healing was quantified using a modified histological assessment scale [50,51,52,53], previously applied in preliminary studies of other chitosan-based formulations [54]. Histological parameters were categorized according to the relevant stages of wound healing: orange for the inflammatory phase (0–7 days), blue for the proliferative phase (3–21 days), and purple for the remodeling phase (2 weeks–1 year). Scores were derived from both H&E and immunohistochemistry slides.

Blood samples collected on days 0 and 22 were used to determine IL-8 concentrations via ELISA, following the manufacturer’s instructions (antibodies-online Inc., Aachen, Germany). Briefly, standards and samples (100 µL each) were incubated in the plate for 120 min at 37 °C (Matrix Orbital Delta plus IKA incubator, Staufen, Germany). Detection reagent A (100 µL) was added and incubated for 60 min at 37 °C, followed by three washes. Detection reagent B (100 µL) was then applied, incubated for 30 min, and washed five times. Substrate solution (90 µL) was added, incubated for 30 min, and the reaction terminated with 50 µL stop solution. Absorbance at 450 nm was measured using a Tecan Sunrise microplate reader (Männedorf, Switzerland). Data were analysed using MyAssays online v. R10.2 software (regression analysis with four-parameter logistic) (MyAssays Ltd., Brighton, UK), and IL-8 concentrations were calculated from the standard curve and expressed in pg/mL.

### 2.7. Statistical Processing of Data

All quantitative data were analysed using SPSS for Windows 17 (IBM Corp., Armonk, NY, USA). Descriptive statistics, including mean ± standard deviation, were calculated for each experimental group. Comparisons between groups were performed using one-way analysis of variance (ANOVA) to assess statistically significant differences. When ANOVA indicated significant differences, post-hoc pairwise comparisons were conducted using Tukey’s test to identify specific group differences. A *p*-value of less than 0.05 was considered statistically significant.

## 3. Results

### 3.1. Nanofibers’ Characterization

**Figure 4:**
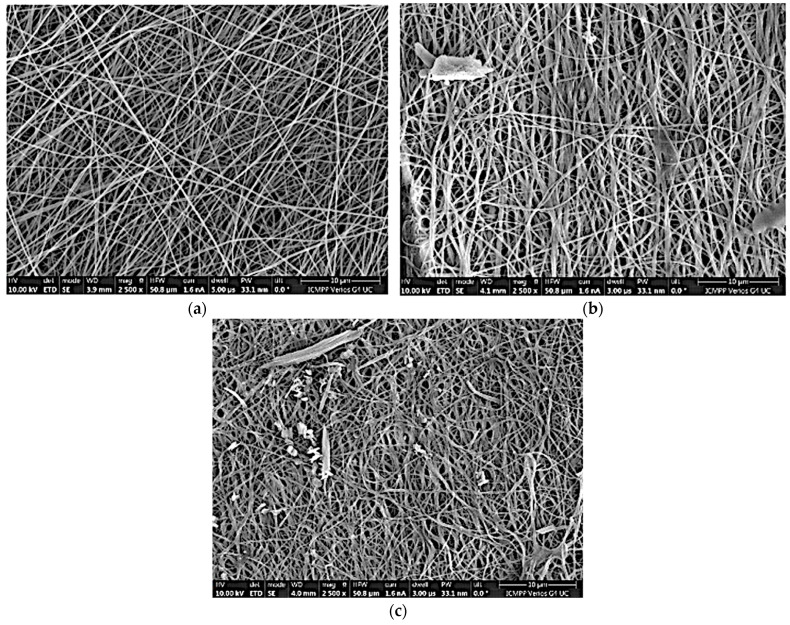
SEM micrographs of (**a**) blank NCC fibers, (**b**) norfloxacin-loaded fibers (NCX), and (**c**) norfloxacin-loaded fibers modified with 2-formylphenylboronic acid (NCXA) (all images acquired at a magnification of 2500×).

The nanoscale morphology, uniformity, and fiber alignment were characterized using SEM. Representative micrographs are presented in Figure 4, showing a homogeneous distribution of fibers with an average diameter of approximately 160 nm.

**Figure 5:**
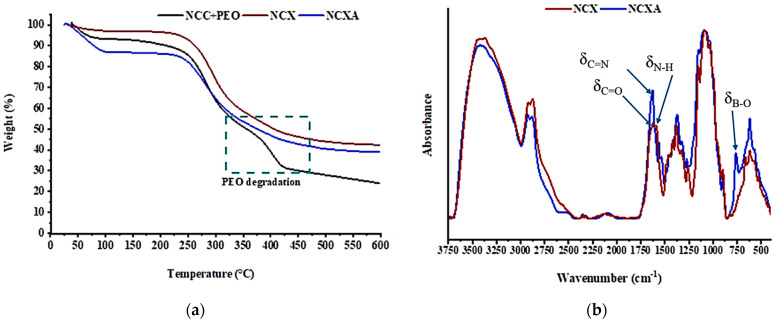
(**a**) Representative thermograms of NCC fibers containing PEO, and NCX and NCXA composite fibers. (**b**) FTIR comparative spectra of NCX and NCXA fibers revealing the formation of imine bonds.

Thermal stability was assessed by thermogravimetric analysis (TGA, Figure 5a). The initial weight loss corresponded to the evaporation of adsorbed water, while a degradation stage associated with PEO was observed up to 400 °C in pre-treated fibers. This degradation phase was absent in fibers following ethanol-mediated PEO removal, confirming the complete elimination of the sacrificial polymer. Overall, the nanofibers exhibited high thermal stability at elevated temperatures.

Beyond demonstrating the thermal stability of the nanofibrous systems, TGA confirmed the effective removal of the sacrificial PEO component following ethanol treatment. This is particularly relevant as it ensures that subsequent biological evaluations are performed on TMC/CS-based matrices without interference from residual electrospinning aids. Moreover, the comparable thermal behaviour of the modified fibers indicates that norfloxacin loading and surface functionalization did not compromise the structural integrity of the nanofibrous platform.

Fiber composition was further verified by FTIR spectroscopy (Figure 5b). The incorporation of norfloxacin was evidenced by characteristic absorption bands, including the NH stretching of the piperazine ring at 1608 cm^−1^, and the B–O bending vibration indicative of the boronic aldehyde moiety at 842 cm^−1^. The complete formation of imine linkages was confirmed by the appearance of the C=N stretching band at 1627 cm^−1^, alongside the disappearance of the carbonyl (C=O) stretching vibration at 1730 cm^−1^ [50,51], demonstrating successful surface modification.

### 3.2. Macroscopical Wound Healing

All animals tolerated the procedures well and demonstrated satisfactory postoperative recovery throughout the study. Progressive wound contraction was observed across all experimental groups, though the rate and extent of healing varied depending on the dressing applied. By day 22, complete epithelialization was achieved in all rats treated with the NCX dressing, indicating its strong wound-healing potential. In contrast, wounds in the V, SS, and NCC groups exhibited near-complete closure, while persistent ulcerations remained in the NCXA group. Analgesic administration was limited to the first five postoperative days, and no unexpected complications were reported, confirming the overall safety and tolerability of all tested interventions.

**Figure 6:**
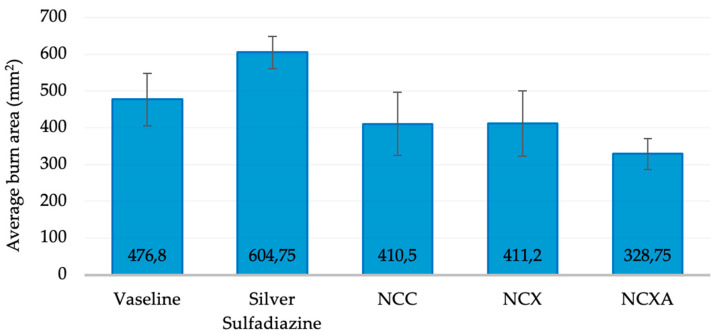
The influence of the dressings used on the burn areas surface at day 22. The values are presented as mean ± standard deviation of mean (S.D.) for 5 rats per group.

Quantitative assessment of wound healing revealed that the greatest reduction in wound area was observed in the NCXA group, followed by the V group (*T*-test, t = 1.09, n − 1 = 19, critical value = 1.729, *p* = 0.05). No statistically significant differences were observed between the NCC and NCX groups, while wounds treated with the SS dressing demonstrated the slowest contraction rate. These results suggest that the physicochemical properties and bioactive components of the dressings may have influenced the healing dynamics (Figure 6). For instance, NCX appears to facilitate rapid epithelialization, possibly through enhanced moisture retention and support for cellular migration, whereas the slower healing observed in the SS group may reflect limited bioactivity or suboptimal interaction with the wound microenvironment (Figure 6).

**Figure 7:**
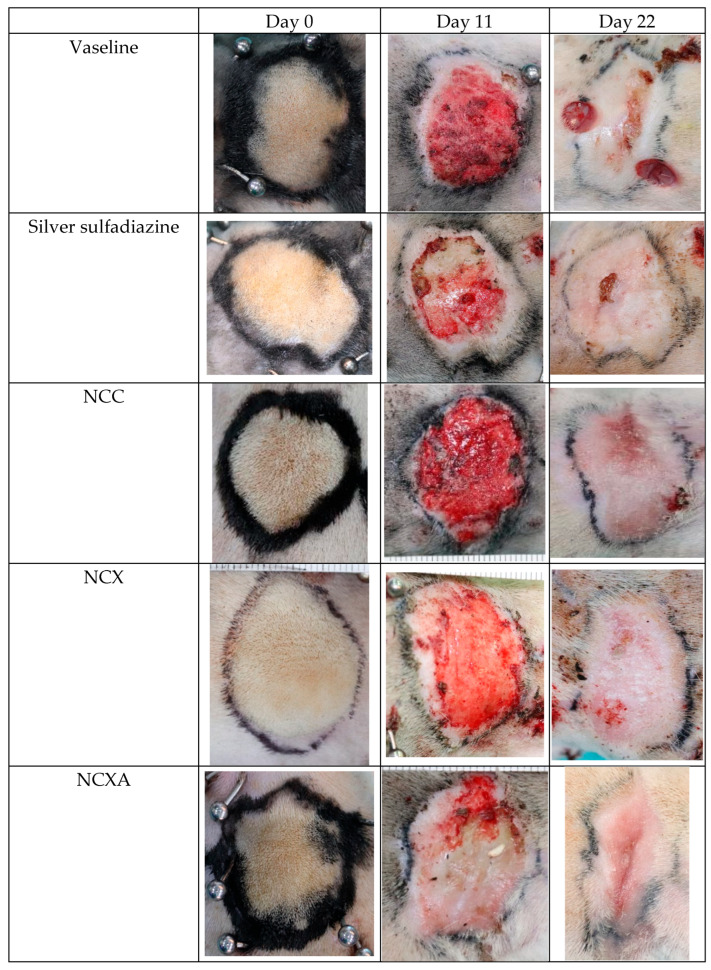
Macroscopical aspect of wound healing evolution in time. Day 0: burn wound with tattooed contour (just outside the burn). Day 11: granulation, centripetal healing. Central fibrin and necrosis in NCXA. Day 22: General good healing rates, despite scarring, scabs and ulcerations. Biopsy sites are present in V group.

At day 0, immediately after burn induction, all experimental wounds exhibited uniform deep dermal damage. In the control animals, the wound was round, slightly depressed, with a whitish appearance, moderate peripheral erythema, and mild oedema. No vesicles or suppuration were observed, and the boundary between the burn area and normal skin was clearly defined. In the nanofiber-treated groups, wounds also had clearly demarcated margins and no signs of infection (Figure 7).

By day 11, control wounds showed persistent crust formation, slight reduction in wound size, and marginal erythema, but overall healing was slow. In the V, NCC and NCX groups, the burn area exhibited reduced edema, a pinkish coloration, and better-defined borders. Specifically, the NCX-treated wounds displayed early formation of shiny epithelial patches, indicating the onset of tissue regeneration. The NCXA group, however, showed delayed contraction, persistent erythema, and patchy epithelial coverage, suggesting a slower transition from the inflammatory to the proliferative phase (Figure 7).

By day 22, NCX-treated wounds achieved near-complete closure, with smooth tissue and small areas of newly formed epithelium, reflecting advanced regeneration and minimal residual inflammation. NCC-treated wounds showed substantial healing, although minor areas of incomplete epithelial coverage remained. Vaseline-treated wounds retained thick, dark crusts with limited healing, while silver sulfadiazine-treated wounds displayed moderate closure with partial epithelialization. NCXA-treated wounds exhibited the slowest progress, with persistent irregularities, delayed epithelial coverage, and signs of ongoing inflammation, highlighting a prolonged inflammatory phase and delayed tissue remodeling (Figure 7).

Macroscopic observations indicate that NCX nanofibers promoted the fastest and most organized healing, NCC fibers supported moderate regeneration, while NCXA fibers delayed progression into the remodeling phase, and both control and commercial dressings showed comparatively slower closure, with solely two applications spaced 11 days apart.

The data indicate that the choice of dressing can significantly affect both the rate and quality of burn wound healing, emphasizing the importance of tailoring wound care strategies to the specific properties of the therapeutic material. These findings provide a foundation for further mechanistic studies aimed at optimizing dressing formulations to maximize tissue regeneration and minimize residual ulceration.

### 3.3. Microscopical Results—Skin Biopsy

#### 3.3.1. Histological Dynamics During Burn Wound Healing

**Figure 8:**
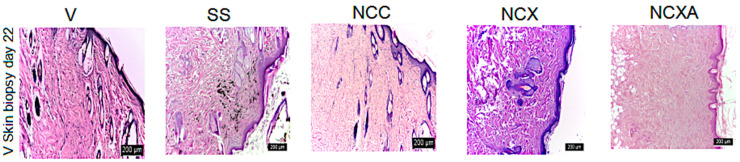
Microscopical wound healing evolution in time. Hematoxylin & Eosin stain × 20. Scale bar = 200 µm.

All groups show variable degrees of healing on day 22, as seen in Figure 8. Wounds treated with V, SS, and NCC dressings exhibited partial epithelial restoration. Histological sections revealed areas of continuous epidermis interspersed with immature granulation tissue, moderate inflammatory cell presence, and less organized collagen deposition.

This pattern aligns with the near-complete wound closure seen macroscopically, reflecting a slightly slower healing trajectory compared to NCX, likely due to less optimal support for cell migration and matrix deposition (Figure 8, Table 1 and Table 2). The thickness and regularity of the epithelium is another supporting factor of the skin quality of wounds treated with NCX.

Intermediate observations on day 11 revealed pronounced alterations in both the epidermal and dermal layers, following the descending order of severity: V, NCC, NCXA, NCX, and SS. Across all groups, the epidermis exhibited a reduced number of layers (1–3), accompanied by dermal ulcerations infiltrated by macrophages and fibroblasts. As anticipated, tattoo pigments remained fixed immediately outside the burn margins (Figure 7 and Figure 8, Table 1 and Table 2). All groups display ongoing neoangiogenesis. Except for NCX, all the other groups display high inflammatory infiltrate and generalized oedema.

Histological examination of the burn wounds provided detailed insights into the cellular and tissue-level processes underpinning the healing observed macroscopically. The dermis displayed extensive angiogenesis, with variable degrees of oedema, connective fiber proliferation, and clusters of fibroblasts. Newly formed hair follicles were sparse, small, and thin-walled, interspersed with degenerated follicles (Figure 7 and Figure 8, Table 1 and Table 2).

In the NCX-treated group, the histology demonstrated complete re-epithelialization with a well-structured epidermis, organized dermal collagen fibers, and extensive fibroblast proliferation. Inflammatory infiltration was minimal, suggesting resolution of the acute inflammatory phase and efficient progression toward tissue remodeling. These findings are fully consistent with the macroscopic observation of complete epithelial coverage, indicating that NCX supports both rapid and high-quality tissue regeneration (Figure 8, Table 1 and Table 2).

The NCXA group, which displayed persistent ulcerations macroscopically, showed histological evidence of incomplete epithelialization, disorganized dermal architecture, sparse fibroblast activity, and ongoing inflammatory infiltration. Collagen fibers were irregular and loosely packed, indicating delayed or impaired tissue regeneration. These cellular-level findings explain the reduced contraction and slower healing observed in this group, highlighting a suboptimal interaction between the dressing material and the wound microenvironment (Figure 8, Table 1 and Table 2).

The correspondence between structural tissue regeneration and surface-level healing emphasizes the critical role of dressing composition in guiding both the rate and quality of burn wound repair. These results underscore the importance of integrating histological evaluation into preclinical wound healing studies to fully understand treatment performance.

#### 3.3.2. Evolution of Immunohistochemical Features in Burn Wounds

**Figure 9:**
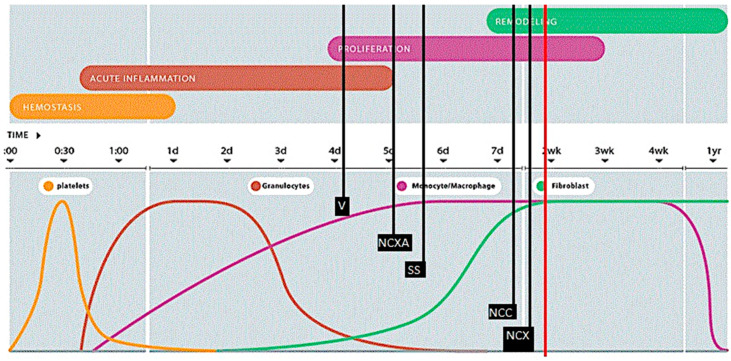
Healing progress of each study group at day 11, relative to the standard theoretical healing timeline (red line), according to relevant cell populations. The proliferative phase should be at a peak, with an equally important presence of fibroblast that triggers remodeling and wound contraction. NCC and NCX are evolving accordingly, while the natural healing is still in a highly inflammatory phase, confirming a deep dermal burn. NCXA and SS biopsies reveal increased inflammatory activity relative to the other groups.

In the V group, TNF-α expression remained elevated, consistent with an active inflammatory phase, while VEGF activity suggested ongoing but comparatively slower angiogenesis. The persistence of necrotic tissue and reduced macrophage activity further indicated a delayed overall healing process in this group (Figure 9, Table 3).

Rats treated with SS exhibited a similar immunohistochemical profile to the V group, except for VEGF levels, which reflected the advanced stage of angiogenesis. Reduced exudate and necrosis relative to V confirmed the superior efficacy of SS, with wound healing progressing according to expected patterns (Figure 9, Table 3).

NCC-treated wounds showed elevated inflammatory markers alongside well-developed functional neo vessels, indicating a favourable healing trajectory. In the NCX group (TMC/CS nanofibers loaded with norfloxacin), the inflammatory response was most pronounced at day 11, accompanied by active collagen deposition, established neovascularization, and a multilayered epidermis (Figure 9, Table 3).

In the NCXA group, the histological profile resembled that of the SS group, though with slightly increased superficial dermal inflammation. The lowest observed levels of cellular differentiation and neovascularization in this group suggested a comparatively slower healing rate (Figure 9, Table 3).

**Figure 10:**
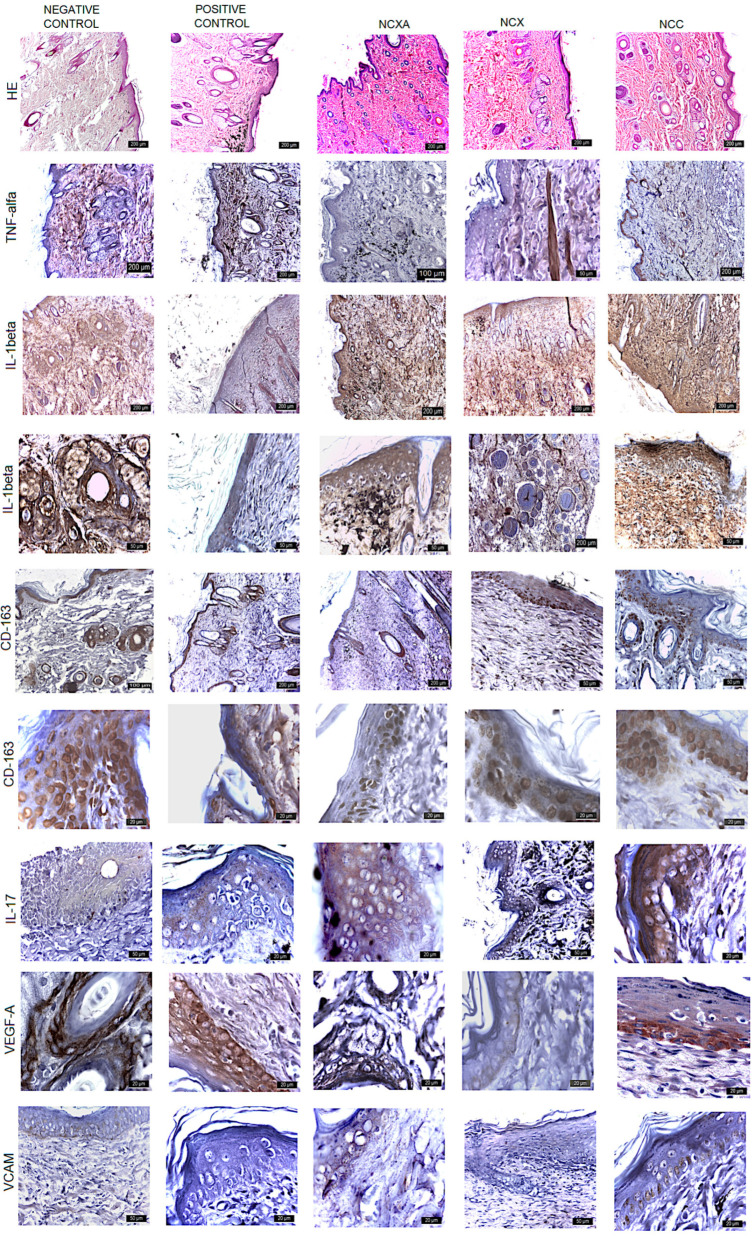
Immunohistochemical staining of the dressing tested at endpoint (day 22).

**Figure 11:**
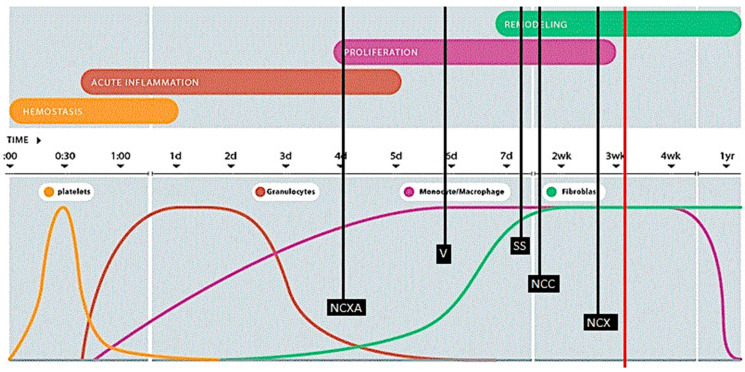
Healing progress of each study group at day 22, relative to the standard theoretical healing timeline (red line), according to relevant cell populations. The proliferative phase should already diminish the intensity, while the fibroblasts dominate. NCX is the only group that follows the natural healing curve of a ‘simple’ wound. The natural healing (V) is slowly advancing through a prolonged inflammatory phase, while SS and NCC show intermediate progress. NCXA is stagnating in a highly inflammatory phase, like a chronic wound.

Persistent elevations of TNF-α, IL-1β, and CD163 in the V-treated wounds indicate ongoing acute inflammation, while sustained VEGF activity at three weeks suggests continued vascular remodeling and a potential shift toward a chronic inflammatory state despite complete epithelialization (Figure 10 and Figure 11 and Table 4).

Moderate generalized inflammation was observed with SS treatment, accompanied by well-preserved cellular differentiation, the highest degree of macroscopic wound contraction, and epidermal thickness and cellularity comparable to the Vaseline group, reflecting effective but not fully optimized tissue repair (Figure 10, Table 4).

Wounds treated with NCC nanofibers exhibited improved skin quality with multilayered epidermis and active collagen synthesis and resorption, indicative of ongoing robust healing (Figure 10, Table 4).

Complete epithelialization was achieved in wounds treated with NCX nanofibers, which also showed the lowest TNF-α, VEGF, and VCAM expression, minimal inflammatory profile, moderate CD163 and IL-17 levels reflecting keratinocyte proliferation and deep dermal regeneration, and fully established angiogenesis. Extensive tissue infiltration, necrotic debris, and pronounced dermal inflammation were evident in wounds treated with NCXA nanofibers, indicating present macrophage activity, thus a severely delayed healing process (Figure 10, Table 3).

#### 3.3.3. Systemic Inflammation Impact

No significant differences were detected in standard haematological parameters, including red blood cell count (RBC), haemoglobin concentration (Hb), and haematocrit (Ht), across the experimental groups. 

**Figure 12:**
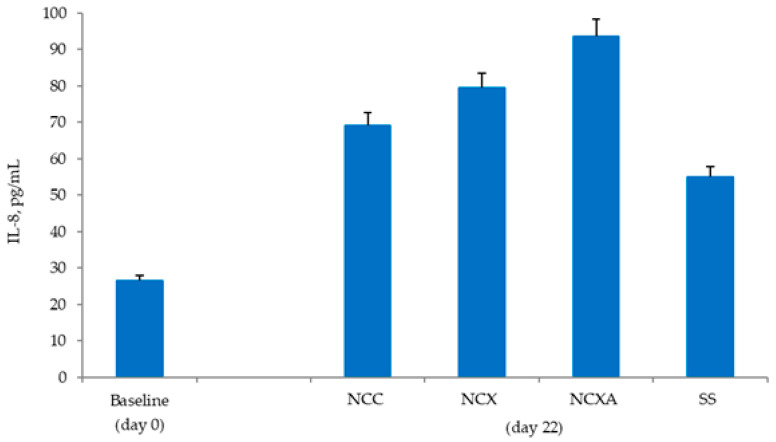
The influence of the dressing used on the blood level of IL-8 in the experiment. The values are presented as mean ± standard deviation of mean (S.D.) for 5 rats per group.

Interestingly, IL-8 was the only serum inflammatory marker to show a statistically significant difference among the groups. Interleukin-8 is a key chemokine involved in the recruitment and activation of neutrophils at sites of tissue injury, playing a central role in the early inflammatory phase of wound healing. Serum IL-8 levels were monitored at multiple time points to capture dynamic changes during the healing process, and the results are summarized in Figure 12. The observed differences in IL-8 suggest that the type of dressing can modulate the systemic inflammatory response, potentially reflecting variations in local wound microenvironment signalling and the progression from inflammation to tissue repair.

These findings emphasize the importance of evaluating both systemic and local immunological responses in preclinical wound healing studies. While standard hematological and biochemical parameters confirm the safety of the tested materials, changes in specific inflammatory mediators like IL-8 provide insight into the interplay between topical interventions and host immune regulation during tissue regeneration.

## 4. Discussions

### 4.1. Overall Therapeutic Performance of Functionalized TMC/CS Nanofibers

Building on our previous studies demonstrating that electrospinning TMC and CS in controlled ratios produces nanofibrous matrices with tunable structural and biological properties [31,34,55,56,57], the present work investigated whether antibiotic loading and surface functionalization could further enhance their therapeutic performance in burn wound healing. Among the formulations evaluated, a CS-dominant composition was selected based on its favorable balance between mechanical integrity, biocompatibility, and biological activity.

The physicochemical characterization confirmed that both norfloxacin incorporation and subsequent elimination were successfully achieved without substantially altering the fibrous architecture. Preservation of the nanofibrous morphology is particularly relevant for wound-healing applications, as electrospun fibers mimic key structural features of the extracellular matrix, providing a highly porous environment that supports cell migration, nutrient exchange, and tissue regeneration. Morphological analyses further confirmed that norfloxacin was predominantly well dispersed throughout the fibrous matrix, while structural characterization demonstrated successful incorporation of all components and complete removal of the sacrificial PEO phase, confirming the effectiveness of the fabrication and modification procedures.

All investigated dressings promoted wound repair in the experimental second-degree burn model; however, marked differences were observed in both the rate and quality of healing. Among the tested formulations, NCX demonstrated the most favorable therapeutic profile, achieving complete wound closure by day 22 and exhibiting superior tissue organization compared with the control groups. Macroscopic observations were consistently supported by histological and immunohistochemical findings, which revealed reduced inflammatory infiltration, advanced re-epithelialization, and a more mature dermal architecture. The concordance between these independent evaluation methods reinforces the robustness of the observed biological effects [3,7,58].

Successful wound healing requires a tightly regulated transition from inflammation to tissue regeneration and remodeling. This process involves coordinated interactions among inflammatory cells, keratinocytes, fibroblasts, endothelial cells, extracellular matrix components, and numerous signaling mediators [59]. The immunohistochemical profile observed in the NCX group suggests that this transition occurred efficiently, resulting in a biological environment favorable for tissue restoration.

Macrophages are key regulators of wound repair because they orchestrate both inflammatory and regenerative responses throughout the different phases of healing [53,60]. During the early stages, they contribute to debris clearance and host defense, whereas later they acquire reparative phenotypes that promote inflammation resolution, extracellular matrix remodeling, angiogenesis, and tissue regeneration [53,61,62,63,64]. In the present study, the expression pattern of CD163, together with the reduced expression of pro-inflammatory cytokines, suggests that NCX promoted a favorable progression toward the remodeling phase. According to the designated scoring system, NCX consistently displayed minimal inflammatory activity, indicating an efficient transition from inflammation to proliferation and remodeling. This interpretation is further supported by the expression profiles of TNF-α and IL-1β. Both cytokines play essential roles during the early inflammatory response by promoting immune-cell recruitment and activating resident cells involved in tissue repair [65,66,67,68,69,70]. However, prolonged elevation of these mediators is frequently associated with delayed healing and chronic inflammatory states [62,71]. The reduced expression of TNF-α and IL-1β observed in NCX-treated wounds at day 22 indicates effective resolution of inflammation and is consistent with the complete epithelialization and improved tissue organization identified histologically [49,51].

Re-epithelialization is a critical event during wound repair and depends on coordinated interactions between keratinocytes, fibroblasts, immune cells, and extracellular matrix components [72]. Keratinocyte migration and proliferation are regulated by multiple cytokines and growth factors present within the wound microenvironment [73,74]. The complete epithelial coverage observed in NCX-treated wounds suggests that the local biological environment generated by this dressing supported efficient epidermal regeneration and restoration of the skin barrier [75]. The angiogenic markers VEGF and VCAM provided additional evidence of advanced healing in the NCX group. Angiogenesis is essential during the proliferative phase because newly formed vessels supply oxygen and nutrients to regenerating tissues. However, sustained expression of angiogenic and endothelial activation markers at later stages often reflects delayed tissue maturation. The lower VEGF and VCAM expression detected in NCX-treated wounds at day 22 suggests that vascular development had largely progressed toward maturation, supporting the histological evidence of advanced tissue remodeling [76,77]. Similarly, the expression pattern of IL-17 may reflect ongoing regenerative activity rather than persistent inflammation. Although traditionally considered a pro-inflammatory cytokine, IL-17 has also been implicated in keratinocyte proliferation, re-epithelialization, and tissue regeneration [65]. Collectively, the observed immunohistochemical profile suggests that norfloxacin-loaded nanofibers promoted a coordinated healing response characterized by controlled inflammation, efficient angiogenesis, progressive tissue maturation, and restoration of functional skin architecture. In contrast, NCXA-treated wounds exhibited a distinct biological behavior. Although wound contraction and epidermal regeneration were evident, complete tissue maturation was not achieved by day 22. Histological examination revealed residual ulceration and delayed dermal organization, while immunohistochemical analyses demonstrated persistently elevated TNF-α, IL-1β, VEGF, and VCAM expression. Together, these findings indicate prolonged inflammatory and angiogenic activity and suggest a delayed transition from the inflammatory and proliferative phases toward tissue remodeling.

Because both NCX and NCXA contained norfloxacin, the differences observed between these formulations are unlikely to be attributable to the antibiotic itself. Instead, they appear to be associated with the additional 2-formylphenylboronic acid surface functionalization. Boronic acid derivatives are known to interact reversibly with cis-diol-containing biomolecules and cell-surface glycoconjugates, potentially influencing cellular recognition processes and local signaling pathways [78,79,80]. While these compounds have attracted considerable interest because of their antimicrobial and antioxidant properties, their effects on wound-healing dynamics remain incompletely understood. The persistent inflammatory and angiogenic profile observed in the NCXA group suggests that this functionalization altered the wound microenvironment in a manner that delayed tissue maturation. Consequently, further optimization of the concentration and surface density of the boronic acid moieties may be required to achieve an optimal balance between antimicrobial activity and regenerative performance.

This hypothesis is further supported by the sustained expression of VEGF and VCAM observed in NCXA-treated wounds at day 22. Whereas VEGF levels generally peak during the proliferative phase and subsequently decline as tissue maturation progresses, elevated late-stage expression is often associated with prolonged granulation tissue formation and delayed remodeling. Similarly, continued VCAM expression indicates ongoing recruitment and activation of inflammatory cells rather than complete resolution of the inflammatory response [81,82,83,84]. These findings suggest that NCXA modified the temporal dynamics of wound healing by prolonging inflammatory and angiogenic events while only partially supporting tissue maturation.

Importantly, the reduction in wound area observed in the NCXA group should not be interpreted as evidence of superior healing. Wound contraction may occur concurrently with persistent inflammation and incomplete tissue maturation and therefore does not necessarily reflect successful tissue regeneration. In contrast, NCX achieved complete epithelialization, reduced inflammatory activity, mature vascularization, and a more organized dermal structure, indicating a more coordinated regenerative process.

The NCC and silver sulfadiazine groups displayed broadly comparable healing patterns, with H&E staining and immunohistochemical analyses revealing similar expression profiles across all investigated markers. In contrast, wounds treated with Vaseline alone followed the expected course of spontaneous repair, characterized by less organized tissue architecture and more pronounced inflammatory activity. Such wounds remain more susceptible to excessive contraction and abnormal scar formation during subsequent remodeling stages.

Serum IL-8 levels reflected the complex systemic response associated with burn healing and likely resulted from the combined effects of tissue injury, extracellular matrix remodeling, collagen synthesis, and the biopsy procedure performed during the study. Although elevated IL-8 concentrations have been associated with enhanced keratinocyte proliferation and wound repair [85,86], and promotion of the transition from the inflammatory to the proliferative phase [87], whereas persistently low IL-8 concentrations are often linked to delayed or impaired repair, this cytokine is not specific to tissue regeneration and should therefore be interpreted within the broader context of the overall inflammatory response. Persistent IL-8 elevation following burn injury has been previously reported [88,89],while increases may also occur in various systemic inflammatory conditions [90,91].

Beyond conventional treatments such as Vaseline gauze and silver sulfadiazine, considerable efforts have recently focused on the development of advanced wound dressings based on electrospun nanofibers and bioactive polymeric scaffolds. Electrospun systems fabricated from polymers such as polycaprolactone (PCL), polylactic acid (PLA), and polyvinyl alcohol (PVA) have demonstrated promising regenerative properties owing to their extracellular matrix-like architecture and their ability to incorporate therapeutic agents [92,93,94,95,96]. However, many synthetic polymers require additional functionalization to provide intrinsic antimicrobial activity or biological responsiveness.

In contrast, chitosan-based systems offer the combined advantages of biocompatibility, hemostatic activity, antimicrobial properties, and immunomodulatory potential [97]. Previous studies have demonstrated that electrospun chitosan-containing nanofibers can support cellular adhesion, proliferation, and tissue regeneration while simultaneously serving as efficient carriers for bioactive compounds [98,99,100]. The findings of the present study are consistent with this growing body of evidence and further demonstrate that localized norfloxacin delivery within a TMC/CS nanofibrous matrix can promote efficient wound repair through improved regulation of inflammation and tissue remodeling [93,100].

More broadly, the present results position NCX within the emerging generation of multifunctional wound dressings that combine structural biomimicry with localized therapeutic delivery. At the same time, the contrasting biological response observed in NCXA highlights the importance of carefully optimizing chemical functionalization strategies, as relatively subtle modifications of the material surface may substantially influence the wound microenvironment and, ultimately, healing outcomes.

Although the selected immunohistochemical markers provided valuable insight into inflammatory, angiogenic, and macrophage-associated responses, they do not fully elucidate the molecular mechanisms underlying the observed therapeutic effects. Future studies incorporating gene-expression analysis, cytokine profiling, macrophage-polarization markers, and signaling-pathway investigations will be necessary to provide a more comprehensive mechanistic understanding of the interactions occurring at the wound–dressing interface.

### 4.2. Study Limitations and Methodological Considerations

Several limitations should be acknowledged when interpreting the present findings. The relatively small sample size (*n* = 5 animals per group), although consistent with exploratory in vivo studies and the principles of animal reduction (3Rs), may have limited the statistical power to detect subtle differences between treatment groups. Therefore, the results should be interpreted as proof-of-concept evidence rather than definitive confirmation of therapeutic superiority. Nevertheless, the biological trends observed were highly consistent across independent evaluation methods, including macroscopic wound assessment, histological examination, immunohistochemical marker expression, and systemic inflammatory profiling. The convergence of these complementary endpoints strengthens confidence in the overall conclusions despite the limited cohort size. Future studies involving larger animal populations and additional validation cohorts will be necessary to further confirm the reproducibility and statistical robustness of the observed effects.

The rat burn model, while widely used and highly reproducible, presents known anatomical and physiological differences compared to human skin, including the presence of the panniculus carnosus and an enhanced intrinsic healing capacity. Consequently, the results should be considered primarily as proof-of-concept findings rather than directly translatable clinical outcomes.

The absence of infection under the controlled experimental conditions employed in this study did not permit a comprehensive in vivo evaluation of antimicrobial efficacy, although both norfloxacin and the selected biomaterials possess well-established antibacterial properties. Likewise, the chosen evaluation time points (days 11 and 22) were selected to represent key stages of the proliferative and remodeling phases of wound healing; however, additional intermediate assessments could provide a more detailed understanding of the early inflammatory response and tissue repair dynamics.

Another limitation concerns the lack of direct drug release characterization. Although controlled local delivery of norfloxacin constitutes one of the principal objectives of the developed nanofibrous dressing, cumulative release profiles, release kinetics, and release mechanism analyses were not assessed in the present study. Owing to the limited availability of the fabricated nanofiber samples and the unavailability of specific reagents required to reproduce the formulations under identical experimental conditions during the revision period, additional release experiments could not be performed. As a result, the release behaviour of norfloxacin from the developed matrices remains to be quantitatively established. Nevertheless, the capacity of chitosan-based nanofibrous systems to provide sustained and controlled drug delivery has been extensively documented in the literature, supporting the rationale behind the present formulation. Future work will therefore focus on a comprehensive investigation of norfloxacin release profiles, kinetic modelling, and the mechanisms governing drug transport within these nanofibrous matrices.

Immunohistochemical assessment was performed using a semi-quantitative scoring approach, which, although widely adopted in similar studies, may be subject to a degree of observer-dependent variability. This limitation was partially mitigated through the simultaneous evaluation of multiple biomarkers and corroborative histological analyses. Finally, while the standardized experimental setup ensured high internal reproducibility, independent validation by other research groups will be important to further confirm the robustness and generalizability of the proposed methodology.

### 4.3. Implications for the Rational Design and Evaluation of Bioengineered Wound Dressings

Assessment of wound healing extends beyond the mere observation of epithelial closure, as rapid wound contraction does not necessarily equate to high-quality tissue regeneration or optimal scar formation. This study highlights the importance of a comprehensive, multi-level evaluation, incorporating macroscopic inspection, histological analysis, and IHC, to accurately interpret healing outcomes. By integrating these methodologies, we were able to identify molecular and cellular markers corresponding to distinct phases of wound repair, thereby distinguishing between effective tissue regeneration and prolonged inflammatory responses.

These findings carry significant implications for the design and evaluation of bioengineered wound dressings. They underscore that dressing performance should not be assessed solely based on closure rates but must also consider tissue quality, cellular differentiation, extracellular matrix organization, and vascular maturation. To our knowledge, this is the first study to apply such a holistic characterization to chitosan-based scaffolds, providing a robust framework for future preclinical and translational investigations in the field of regenerative wound care.

## 5. Conclusions

The present study demonstrated that electrospun TMC/CS nanofibers represent a promising platform for advanced wound dressing applications. Among the investigated formulations, norfloxacin-loaded nanofibers (NCX) showed the most favourable therapeutic profile, promoting complete epithelialization, reduced inflammatory activity, and improved tissue organization in a rat burn model.

In contrast, additional modification with 2-formylphenylboronic acid (NCXA) was associated with prolonged inflammation and delayed tissue remodeling despite continued wound contraction. The combined macroscopic, histological, immunohistochemical, and systemic assessments highlighted the importance of evaluating both wound closure and tissue quality when assessing dressing performance. These findings support the potential of norfloxacin-loaded TMC/CS nanofibers as multifunctional wound-healing materials.

Further studies are required to optimize the boronic acid modification strategy, investigate the mechanisms responsible for the delayed healing response observed in NCXA, evaluate norfloxacin release kinetics, and validate the long-term efficacy and safety of these systems in additional preclinical models.

## Figures and Tables

**Table 1:** Scoring of wound parameters on day 11. Wound scores were assigned as follows: 3 = intense, 2 = moderate, 1 = low, and 0 = not applicable. Color coding indicates the corresponding phase of wound healing: orange = inflammatory phase, blue = proliferative phase, and purple = remodeling phase.

	Wound Healing Criteria	V	SS	NCC	NCX	NCXA
	Congestion	1	2	1	1	2
	Inflammatory oedema	2	2	2	2	3
	Fibrinous exudate	2	1	1	1	2
	Leucocyte infiltration (neutrophils, macrophage, lymphocyte, histiocyte)	2	2	2	1	2
	Necrosis zone separation and/or resorption	3	2	2	3	2
	Cell differentiation (fibroblasts, endothelial cells)	2	2	2	2	2
	Neoangiogenesis	3	3	3	3	1
	Epithelialization	2	1	1	2	1

**Table 2:** Scoring of parameters at day 22. Wound scores were assigned as follows: 3 = intense, 2 = moderate, 1 = low, and 0 = not applicable. Color coding indicates the corresponding phase of wound healing: orange = inflammatory phase, blue = proliferative phase, and purple = remodeling phase.

	Wound Healing Criteria	V	SS	NCC	NCX	NCXA
	Congestion	0	0	0	0	0
	Inflammatory oedema	0	0	0	0	1
	Fibrinous exudate	0	1	1	0	2
	Leucocyte infiltration (neutrophils, macrophage, lymphocyte, histiocyte)	2	2	2	1	3
	Necrosis zone separation and/or resorption	1	2	2	1	3
	Cell differentiation (fibroblasts, endothelial cells)	3	3	3	3	2
	Neoangiogenesis	2	2	2	2	3
	Epithelialization	3	3	3	3	2

**Table 3:** IHC Markers expression at Day 11 (+++ is high, ++ is moderate, + is low).

IHC Marker	V	SS	NCXA	NCX	NCC
TNF-α	++	++	++	+++	+++
IL-1β	+++	+++	+++	++	+++
CD163	++	++	++	+++	++
IL-17	+	++	++	++	++
VEGF	+++	+	++	+	++
VCAM	+	+	+	+	++

**Table 4:** IHC markers expression at Day 22 (+++ is high, ++ is moderate, + is low, 0 is mostly absent).

Marker IHC	V	SS	NCXA	NCX	NCC
TNF-α	+++	++	+	0	+
IL-1β	+++	++	+++	++	+++
CD163	+++	++	++	++	++
IL-17	+	++	+++	++	+++
VEGF	+++	+++	++	+	+++
VCAM	+	+	++	+	+++

## Data Availability

The data supporting the findings of this study are available from the corresponding author upon reasonable request. The data are not publicly available due to institutional data management policies.
